# Clinicians’ Perspectives on a Web-Based System for Routine Outcome Monitoring in Old-Age Psychiatry in the Netherlands

**DOI:** 10.2196/jmir.1937

**Published:** 2012-05-30

**Authors:** Marjolein A Veerbeek, Richard C Oude Voshaar, Anne Margriet Pot

**Affiliations:** ^1^Netherlands Insitute of Mental Health and AddictionUtrechtNetherlands; ^2^UMC GroningenUniversity Center of PsychiatryGroningenNetherlands; ^3^Department of Clinical PsychologyVU UniversityAmsterdamNetherlands

**Keywords:** Quality of care, health care, routine outcome monitoring, Web-based monitoring, clinicians, feedback

## Abstract

**Background:**

In health care, the use of physical parameters to monitor physical disease progress is common. In mental health care, the periodic measurement of a client’s functioning during treatment, or routine outcome monitoring, has recently become important. Online delivery of questionnaires has the potential to reduce clinicians’ resistance to the implementation of routine outcome monitoring. Online delivery enables clinicians to receive results on a questionnaire in a graphic directly after data entry. This gives them insight into the progress of a client at a single glance.

**Objective:**

To explore clinicians’ perspectives on a routine outcome monitoring procedure where questionnaires and feedback on scores were delivered online. Questionnaires could also be filled out on paper and then entered into the online system by a research assistant.

**Methods:**

In 2009 we sent an online survey, consisting of five yes-or-no questions and six open-ended questions, to all clinicians in the 14 mental health care organizations working with the routine outcome monitoring system in the Netherlands. Of the 172 clinicians contacted, 80 (47%) opened the link and 70 of these 80 (88%) clinicians completed the survey.

**Results:**

Clinicians seldom used the graphical feedback from the Web-based system, which indicates that direct feedback on scores did not enhance the implementation of routine outcome monitoring. Integration into the electronic patient record and more training on interpretation and implementation of feedback in daily practice were seen as the primary points for further improvement. It was mainly the availability of a research assistant that made the routine outcome monitoring procedure feasible.

**Conclusions:**

Without a research assistant and training in the interpretation of outcomes, software programs alone cannot ensure effective implementation of monitoring activities in everyday practice.

## Introduction

Regular monitoring of clients with chronic physical diseases using specific outcome measures—for example glucose levels in diabetes or blood pressure in patients with heart failure—is common in health care [1-3]. In mental health care, the periodic measurement of clients’ functioning during treatment, or routine outcome monitoring, has only recently become important [4-7]. By providing regular information to both the professional and the client on the course and severity of symptoms during treatment, routine outcome monitoring is assumed to improve informed decision making and therefore quality of care. Well-timed information on the severity and course of symptoms during treatment is an integral part of routine outcome monitoring [8,9]. Increased workload due to systematic data collection, such as filling out questionnaires multiple times, can easily lead to resistance by clinicians [10]. Therefore, the additional burden of data collection should balance the utility of these data from the clinician’s perspective.

Online delivery of questionnaires may facilitate routine outcome monitoring by (1) reducing the logistic burden, (2) being able to provide feedback directly to clinicians, which is an extension of the paper-and-pencil method, and (3) reducing the amount of missing data, because one may not proceed without answering all questions [6,10,11]. However, previous research has suggested that attention should be paid to cross-validation between electronic and paper-and-pencil versions of the questionnaires, as psychometric properties may change [12-14] and Internet-based surveys do not automatically translate into higher response rates than with paper-based versions [15-19].

To stimulate the use of routine outcome monitoring in old-age psychiatry in the Netherlands, we introduced the Mental Health Care Monitor Older Adults (MEMO) system. This Web-based system was designed to deliver the benefits mentioned above and contains Web-based versions of questionnaires, automatically calculated sum scores, graphics presenting the symptom course over time, and reminders to clinicians of when follow-up assessments are needed. Depending on their own individual preferences, clinicians could also fill out the questionnaires on paper and a research assistant would then enter the data into the Web-based system. The present study explored the acceptability of using a Web-based routine outcome monitoring system in old-age psychiatry from the perspective of the clinician.

## Methods

### Description of MEMO

MEMO was started in 2008 for a period of 5 years. During information meetings, managers received extensive information about the procedure of MEMO. After these meetings, 14 of the 41 mental health care organizations throughout the Netherlands applied to participate in MEMO. All clients referred by their general practitioner to these 14 organizations and who progressed to treatment after intake at an outpatient clinic for old-age psychiatry were monitored. The only exclusion criterion was a primary diagnosis of a cognitive disorder. Data were collected at fixed intervals and included diagnostic variables, measures of mental and social functioning, treatment characteristics, and client satisfaction (see Table 1 [20-23]).

Before the introduction of MEMO, outcomes were not measured in such a structured and uniform way in old-age psychiatry in the Netherlands. To ensure high-quality data collection, funding was provided to organizations to employ a research assistant. We also provided annual training sessions for the use of the Health of the Nation Outcome Scales for older adults (HoNOS 65+) and the Web-based system (see below).

**Table 1:** Measurement overview of Mental Health Care Monitor Older Adults (MEMO).

Measure	Rater	Time point			
		Intake	4 months	8 months	12 months/end of treatment
Demographic variables	MHP^a^	Yes	No	No	No
DSM-IV-TR^b ^classification	MHP	Yes	No	No	No
HoNOS 65+^c ^(mental and social functioning)	MHP	Yes	Yes	Yes	Yes
GDS-15^d ^(severity of depressive symptoms)^e^	Client	Yes	Yes	Yes	Yes
Type of treatment^f^	MHP	No	Yes	Yes	Yes
Life events	MHP	No	No	No	Yes
MHCCT^g ^(client satisfaction)	Client	No	No	No	Yes

^a ^Mental health care professional.

^b ^Diagnosis according to the *Diagnostic and Statistical Manual of Mental Disorders*, 4th edition, text revision [20].

^c ^Health of the Nation Outcome Scales for older adults [21].

^d ^Geriatric Depression Scale 15-item version [22].

^e ^To be filled out only by clients with a primary diagnosis of a mood disorder.

^f ^Categories are based on the Dutch reimbursement categorization: psychological therapy, supportive counseling, activating techniques, psychopharmaceutical treatment, relation or system therapy, and electroconvulsive therapy.

^g ^Mental Health Care Client Thermometer [23].

As clients neither were subjected to interventions nor had to obey behavioral rules for MEMO, the medical ethics committee Medisch-ethische Toetsingscommissie instellingen Geestelijke Gezondheidszorg decided that the Dutch Medical Research Involving Human Subjects Act was not applicable and the study did not require ethics approval. Nevertheless, all clients were informed about the scientific purposes of MEMO, and clients who did not agree to the use of their anonymous data were excluded.

### Web-Based Data Collection

NetQuestionnaires Netherlands (Utrecht, the Netherlands; www.netq.nl) developed our Web-based system for both data acquisition and processing (sum scores and graphical presentation of symptom course).

To safeguard the data collected, we observed the Data Protection Act and ensured confidentiality through the use of an HTTPS connection, which secured the communication on the Internet, as well as a login procedure and a separate environment for each organization, which prevented the exchange of client data between organizations [24].

**Figure 1:**
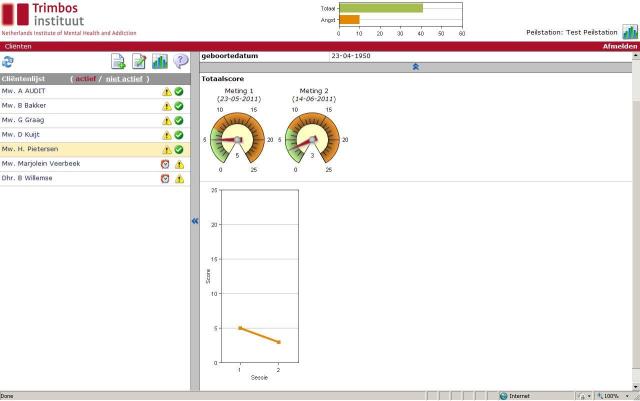
Example of the graphical feedback generated by the Web-based system to show clinicians the progress of their client during treatment. Clarification of Dutch language terms: *Totaalscore* = sum score (at a particular questionnaire); *Meting 1* = sum score at intake; *Meting 2* = sum score at the second moment in time; *Score* (y-axis) = sum score; *Sessie* (x-axis) = session (1 = intake, 2 = second moment in time). Clarification of symbols: exclamation mark = questionnaires have to be filled out for this particular client; check mark = the client has ended treatment; alarm clock = client has been monitored for 1 year and therefore monitoring can be ended.

After a client was added to the system, based on the diagnosis, the questionnaires that had to be filled out at baseline were shown and could be filled out immediately online. During follow-up, the system tracked each client and signaled every 4 months, up to 12 months, by email alerts to both clinicians and research assistants when new questionnaires had to be completed. Immediately after data entry, the system generated a graphic visualizing the progress of the client and thereby giving feedback to the clinician. For example, the feedback in Figure 1 shows that the client had a score of 5 at intake and improved after 4 months to a score of 3.

For clinicians who preferred to use paper-and-pencil forms, feedback was delayed until the research assistant had entered the data into the system. The participating organizations asked clients to fill out their questionnaires on paper.

### Evaluation Questionnaire

#### Measurement

To evaluate the MEMO procedure, we emailed an online questionnaire to all 172 clinicians who were authorized to use the Web-based system after 1 year of data collection. They were asked to help improve the procedure of MEMO by filling out the questionnaire, which would take 10 minutes at most. Responses would not be communicated to their organization or manager. To avoid duplicate entries the software did not allow clinicians to open the questionnaire more than once. In addition to general information (organization, sex, age, profession, and work experience), the questionnaire consisted of 5 items that could be answered with either yes or no: (1) Do you think the MEMO procedure is feasible? (2) Do you use the graphical feedback from the system? If yes, the following questions were displayed: (3) Do you think the graphical feedback is relevant to your work? (4) Based on the graphical feedback, did you change the treatment plan for a specific client? (5) Do you think this type of feedback should be continued? Each question was accompanied by an open-ended question that gave them an opportunity to explain their answer. Next, clinicians were asked to grade the graphical feedback (on a scale of 1 to 10). The final question for all clinicians was “Do you have any suggestions for data collection and giving feedback in mental health care for older adults?” The questions were spread over 4 pages: (1) general information, (2) feasibility questions, (3) questions on the graphical feedback, (4) suggestions for data collection. Clinicians were able to review and change their answers in the course of completing the questionnaire. Responses were automatically captured in a database. To improve the response rate, (1) contrary to the yes-or-no questions, the open-ended questions were not mandatory to prevent premature discontinuation, (2) clinicians received a reminder after 1 month, and (3) a gift voucher of €25 was raffled off among the respondents in each organization.

#### Analysis

Qualitative data analysis was performed on the answers to the open-ended questions. The contents of the answers were labeled (by MV). These labels were data driven. When coding was complete, the frequencies of the codes were analyzed.

## Results

Of the 172 clinicians, 80 (47%) opened the link to the evaluation questionnaire; 70 of these 80 (88%) clinicians completed the questionnaire (n = 41, 59% female; mean age 47.5, range 27–64 years; mean work experience 22, range 1–44 years). Participating clinicians were nurses (n = 38, 54%), psychologists (n = 24, 34%), psychiatrists or geriatricians (n = 5, 7%), and social workers (n = 3, 4%). Characteristics of nonresponders are unknown.

Although 54 of the 70 (77%) clinicians considered the procedures of MEMO to be feasible, only 11 of 70 (16%) actually used the graphical feedback (Table 2). The 11 clinicians who used the feedback considered that it was relevant to everyday practice and that it should be continued. They rated the quality of the feedback with a score of 7 (range 5–8). Clinicians who did not use the feedback gave the following reasons: no time (n = 8), forgot how to use the feedback (n = 5), did not understand graphical feedback (n = 3), and had recently joined the team and therefore had not yet had the opportunity to measure clients multiple times (n = 4).

**Table 2:** Clinicians’ answers to the yes-or-no questions on the feasibility of Mental Health Care Monitor Older Adults (MEMO) and the use of feedback from the Web-based system.

Question		n	Positive answer (yes)	
			n	%
1	Procedure MEMO feasible	70	54	77%
2	Use of graphical feedback	70	11	16%
3	Graphical feedback relevant	11	10	91%
4	Change of treatment plan	11	4	36%
5	Continuation of graphical feedback	11	11	100%

Of 70 clinicians, 21 (30%) gave further suggestions, and 8 of these 21 (38%) suggested that the Web-based system could be improved by incorporating the system within the electronic patient record, through which alerts and graphics are seen directly in the record. The additional login procedure besides the electronic patient record was thought to be the main barrier to the use of the Web-based system. Instead, they filled out the questionnaires on paper. Of the 21 clinicians, 6 (29%) suggested that data collection be extended to other instruments beyond the HoNOS 65+ to obtain more detailed information. Of the 21 clinicians, 5 (24%) suggested that more attention should be paid to the implementation of routine outcome monitoring in everyday practice. Remarkably, only two comments about increased workload were given.

## Discussion

Although the majority of clinicians considered the procedure of MEMO to be feasible, only a few actually used the graphical feedback from the Web-based system. The most common suggestion given to enhance the use of the feedback was to incorporate the Web-based system into the electronic patient record, so that the scores and graphics of their clients would be more easily accessible. This means that previous findings that software programs support direct feedback and monitor activities in clinical practice [6] hold true only if these programs are integrated into the electronic patient record.

As we did not integrate our Web-based system into the electronic patient record, it is likely that the availability of a research assistant made our routine outcome monitoring procedures feasible. This is in line with the findings of a previous study on younger adults, which showed that routine outcome monitoring is highly feasible when supported by trained assistants [7]. However, in that project, the routine outcome monitoring assistants carried out all measurements and reported to the clinicians by letter. In our study, clinicians performed measurements themselves and the research assistants did not report results. The research assistants only entered data into the Web-based system to enable clinicians to see the graphical feedback. Recent research showed that when female cancer survivors were given a choice between filling out a questionnaire online and filling out a paper version of the questionnaire, they preferred to fill out the paper version. When they were not given the choice, their response rates were similar for both the Web-based and paper-based questionnaire [25]. This provides further evidence that it was the availability of a research assistant that made our routine outcome monitoring procedure feasible, since clinicians had a choice and probably preferred to fill out the questionnaires on paper. However, the added value of our research assistants was probably much greater than simple data entry for the following reasons: (1) they had an overview of the entire project, and (2) they were easily accessible to clinicians for support using the Web-based system. Finally, they reminded clinicians to follow up measurements before passing the deadline. Therefore, even when clinicians did not have the opportunity to fill out questionnaires on paper, the support of a research assistant was critical to the implementation of the routine outcome monitoring and is probably the reason that in our study only two comments on the increased workload were made. Previously reported barriers to routine outcome monitoring implementation from the clinician’s perspective included (1) the opinion that the expert knows best, (2) outcome measurement undermining clinical expertise and violating the privacy of the therapy dyad, (3) increased workload, and (4) fear of evaluation of their practice [6,7,10,26,27]. None of these reasons were mentioned in our survey. Instead, a lack of knowledge of how to implement outcome measures in day-to-day clinical practice appeared to be another barrier. Future research should investigate whether more training enhances the use of direct feedback in clinical practice. After succesful implementation of MEMO, results will provide a proxy of quality of care. Additional measures of routine outcome monitoring will be needed to indicate quality of care in old-age psychiatry.

### Limitations

First, the overall response rate (70/172, 41%) is rather low. Nevertheless, most nonresponders (92/172, 54%) did not open the link and therefore were not aware of the content of the survey. As 88% (70/80) of the clinicians who opened the link also filled out the questionnaire, it seems unlikely that the response rate is related to the content of the questionnaire. Second, as the explanatory items were not mandatory, some clinicians may have too easily skipped some of these questions. Therefore, reasons for not using the feedback or suggestions to improve the procedure of MEMO may have been missed. Third, open-ended questions were labeled by one person. Since the questions were structured and the clinicians’ answers were brief, it is likely that having a second person coding would have led to the same results. Fourth, organizations voluntarily applied for participation in MEMO, which could imply that participating clinicians were more in favor of using a Web-based system than were clinicians in organizations that did not participate. However, this seems unlikely to have been the case, because it was the managers who made the decision to participate in MEMO and not the clinicians themselves.

### Conclusion

The availability of a research assistant seems to enhance the acceptability of routine outcome monitoring to clinicians. Integration of routine outcome monitoring software into the electronic patient record and more training on how to implement direct feedback in daily practice would make the Web-based system easier to use for clinicians.

## Abbreviations

HoNOS 65+: Health of the Nation Outcome Scales for older adults
MEMO: Mental Health Care Monitor Older Adults

## References

[1] Wu RC, Delgado D, Costigan J, Maciver J, Ross H (2005). Pilot study of an Internet patient-physician communication tool for heart failure disease management. J Med Internet Res.

[2] Mazzi CP, Kidd M (2002). A framework for the evaluation of Internet-based diabetes management. J Med Internet Res.

[3] Gillespie G (2000). Deploying an I.T. cure for chronic diseases. Health Data Manag.

[4] van der Krieke L, Emerencia AC, Aiello M, Sytema S (2012). Usability evaluation of a web-based support system for people with a schizophrenia diagnosis. J Med Internet Res.

[5] Gilbody SM, House AO, Sheldon TA (2003). Outcome measures and needs assessment tools for schizophrenia and related disorders. Cochrane Database Syst Rev.

[6] Lambert MJ, Harmon C, Slade K, Whipple JL, Hawkins EJ (2005). Providing feedback to psychotherapists on their patients' progress: clinical results and practice suggestions. J Clin Psychol.

[7] de Beurs E, den Hollander-Gijsman ME, van Rood YR, van der Wee NJ, Giltay EJ, van Noorden MS, van der Lem R, van Fenema E, Zitman FG (2011). Routine outcome monitoring in the Netherlands: practical experiences with a web-based strategy for the assessment of treatment outcome in clinical practice. Clin Psychol Psychother.

[8] Zwanepol F, de Groot W (2008). [Implementation of routine outcome monitoring: experiences with clients with mood and anxiety disorders]. Maandblad Geestelijke Volksgezondheid.

[9] Srebnik D, Hendryx M, Stevenson J, Caverly S, Dyck DG, Cauce AM (1997). Development of outcome indicators for monitoring the quality of public mental health care. Psychiatr Serv.

[10] Madan A, Borckardt JJ, Connell A, Book SB, Campbell S, Gwynette MF, Wimberly LA, Wagner M, Weinstein B, McLeod-Bryant S, Cooney H, Herbert J (2010). Routine assessment of patient-reported outcomes in behavioral health: room for improvement. Qual Manag Health Care.

[11] de Nooijer J, de Vries NK (2007). Monitoring health risk behavior of Dutch adolescents and the development of health promoting policies and activities: the E-MOVO project. Health Promot Int.

[12] Shervin N, Dorrwachter J, Bragdon CR, Shervin D, Zurakowski D, Malchau H (2011). Comparison of paper and computer-based questionnaire modes for measuring health outcomes in patients undergoing total hip arthroplasty. J Bone Joint Surg Am.

[13] Touvier M, Méjean C, Kesse-Guyot E, Pollet C, Malon A, Castetbon K, Hercberg S (2010). Comparison between web-based and paper versions of a self-administered anthropometric questionnaire. Eur J Epidemiol.

[14] Whitehead L (2011). Methodological issues in Internet-mediated research: a randomized comparison of internet versus mailed questionnaires. J Med Internet Res.

[15] Leece P, Bhandari M, Sprague S, Swiontkowski MF, Schemitsch EH, Tornetta P, Devereaux PJ, Guyatt GH (2004). Internet versus mailed questionnaires: a controlled comparison (2). J Med Internet Res.

[16] McMahon SR, Iwamoto M, Massoudi MS, Yusuf HR, Stevenson JM, David F, Chu SY, Pickering LK (2003). Comparison of e-mail, fax, and postal surveys of pediatricians. Pediatrics.

[17] Kim HL, Gerber GS, Patel RV, Hollowell CM, Bales GT (2001). Practice patterns in the treatment of female urinary incontinence: a postal and internet survey. Urology.

[18] Harewood GC, Yacavone RF, Locke GR, Wiersema MJ (2001). Prospective comparison of endoscopy patient satisfaction surveys: e-mail versus standard mail versus telephone. Am J Gastroenterol.

[19] Raziano DB, Jayadevappa R, Valenzula D, Weiner M, Lavizzo-Mourey R (2001). E-mail versus conventional postal mail survey of geriatric chiefs. Gerontologist.

[20] American Psychiatric Association (2000). Diagnostic and Statistical Manual of Mental Disorders:DSM-IV-TR. 4th edition, text revision.

[21] Burns A, Beevor A, Lelliott P, Wing J, Blakey A, Orrell M, Mulinga J, Hadden S (1999). Health of the Nation Outcome Scales for elderly people (HoNOS 65+). Br J Psychiatry.

[22] Sheikh RL, Yesavage JA (1986). Geriatric Depression Scale (GDS): recent evidence and development of a shorter version. Clin Gerontol.

[23] Kok I, Mulder E (2005). [Client Satisfaction in Mental Health Care: Manual for Different Client Thermometers in Use].

[24] Kelly G, McKenzie B (2002). Security, privacy, and confidentiality issues on the Internet. J Med Internet Res.

[25] van den Berg MH, Overbeek A, van der Pal HJ, Versluys AB, Bresters D, van Leeuwen FE, Lambalk CB, Kaspers GJ, van Dulmen-den Broeder E (2011). Using web-based and paper-based questionnaires for collecting data on fertility issues among female childhood cancer survivors: differences in response characteristics. J Med Internet Res.

[26] McGrath BM, Tempier RP (2003). Implementing quality management in psychiatry: from theory to practice--shifting focus from process to outcome. Can J Psychiatry.

[27] Robertson L, Smith M, Castle D, Tannenbaum D (2006). Using the Internet to enhance the treatment of depression. Australas Psychiatry.

